# Supplementary material for: Phytotoxin production in Aspergillus terreus is regulated by independent environmental signals
Source: eLife. 2015 Jul 14;4:e07861. doi: 10.7554/eLife.07861 (PMC4528345; doi:10.7554/eLife.07861)
Supplement: Figure 4—source data 1. — DOI: http://dx.doi.org/10.7554/eLife.07861.010 [file elife-07861-fig4-data1.doc]

Figure 4 – Source Data 1. Genotypes of strains used in the study.

| Strain | Genotype | Reference |
| --- | --- | --- |
| *Aspergillus niger* FGSC A1144 | wild type | FGSC; Kansas City; USA |
| *Aspergillus nidulans* FGSC A4 | wild type | FGSC; Kansas City; USA |
| *Aspergillus fumigatus* ATCC 46645 | wild type | ATCC; Manassas; USA |
| *Aspergillus fumigatus* FGSC A1163 (alternate name: CBS 144.89) | wild type | FGSC; Kansas City; USA |
| *Fusarium graminearum* FGSC 9975 | wild type | FGSC; Kansas City; USA |
| *Aspergillus terreus* FGSC A1156  (alternate name: NIH 2624) | wild type | FGSC; Kansas City; USA |
| *Aspergillus terreus* SBUG844 | wild type | HKI; Jena; Germany |
| SBUG844Δ*akuB* | Δ*akuB*::*hph* | Gressler *et al.*, 2011 |
| SBUG844Δ*akuB*Δ*terA* | Δ*akuB*::*hph*; Δ*terA*::*ptrA* | Zaehle *et al*., 2014 |
| SBUG844Δ*akuB*Δ*terR* | Δ*akuB*::*hph*; Δ*terR*::*ptrA* | Zaehle *et al*., 2014 |
| SBUG844_P*terA*:*lacZ* | *ptrA*, P*terA*:*lacZ*:*trpC*^T^ | This study. |
| SBUG844_AnP*gpdA*:*atfA* | *hph*, AnP*gpdA*:*atfA:atfA*^T^ | This study. |
| SBUG844Δ*akuB*Δ*cpcA* | Δ*akuB*::*hph*; Δ*cpcA*::*ptrA* | This study. |
| SBUG844Δ*akuB*Δ*rhbA* | Δ*akuB*::*hph*; Δ*rhbA*::*ptrA* | This study. |
| SBUG844Δ*akuB*Δ*areA* | Δ*akuB*::*hph*; Δ*areA*::*ptrA* | This study. |
| SBUG844Δ*akuB*Δ*areA/areA*^c^ | Δ*akuB*::*hph*; Δ*areA*::*areA*^C^_A1156_ | This study. |
| SBUG844Δ*akuB*Δ*atfA* | Δ*akuB*::*hph*; Δ*atfA*::*ptrA* | This study. |
| SBUG844Δ*akuB*Δ*atfA/atfA*^C^ | Δ*akuB*::*hph*; Δ*atfA*::*atfA*^C^, *ble* | This study. |
| SBUG844Δ*akuB*Δ*areA*Δ*atfA* | Δ*akuB*::*hph*; Δ*areA*::*ptrA;* Δ*atfA*::*ble* | This study. |
| SBUG844Δ*akuB*Δ*areA*Δ*atfA*::AnP*gpdA*:*terR* | Δ*akuB*::*hph*; Δ*areA*::*ptrA;* Δ*atfA*::AnP*gpdA*:*terR*:*terR*^T^, *ble* | This study. |
| SBUG844Δ*akuB*Δ*hapX* | Δ*akuB*::*hph*; Δ*hapX*::*ptrA* | This study. |
| SBUG844Δ*akuB*Δ*hapX/*An*hapX*^C^ | Δ*akuB*::*hph*; Δ*hapX*::*hapX*_An_^C^*, ble* | This study. |
| SBUG844Δ*akuB*Δ*sreA* | Δ*akuB*::*hph*; Δ*sreA*::*ptrA* | This study. |
| SBUG844Δ*akuB*Δ*sreA*:*sreA*^C^ | Δ*akuB*::*hph*; Δ*sreA*::*ptrA; sreA*, *ble* | This study. |
| SBUG844Δ*akuB*Δ*sidA* | Δ*akuB*::*hph*; Δ*sidA*::*ptrA* | This study. |
| SBUG844Δ*akuB*Δ*sidA/sidA*^C^ | Δ*akuB*::*hph*; Δ*sidA*::*sidA*^C^*, ble* | This study. |
| SBUG844Δ*akuB*Δ*terA*Δ*sidA* | Δ*akuB*::*hph*; Δ*terA*::*ptrA;* Δ*sidA*::*ble* | This study. |
|  |  |  |
