# Supplementary material for: Phytotoxin production in Aspergillus terreus is regulated by independent environmental signals
Source: eLife. 2015 Jul 14;4:e07861. doi: 10.7554/eLife.07861 (PMC4528345; doi:10.7554/eLife.07861)
Supplement: Figure 4—source data 2. — DOI: http://dx.doi.org/10.7554/eLife.07861.011 [file elife-07861-fig4-data2.doc]

**Figure 4 – Source** Data 2. List of oligo nucleotides used in the study.

| no. | name | 5´-3´sequence |
| --- | --- | --- |
| P1 | qPCR_actA_f2 | GTCTGGAGAGCGGTGGTATC |
| P2 | qPCR_actA_r2 | AGGAGCGATGATCTTGACCT |
| P3 | qPCR_enoA_f2 | GGAGGCCTGGAGCTACTTCT |
| P4 | qPCR_enoA_r2 | CAGTTGTCGGCATAGGAGTC |
| P5 | qPCR_areA_f | ATCCCACTCAGGTGCTGAA |
| P6 | qPCR_areA_r | ACTGCATGCCAGTGGACTC |
| P7 | qPCR_atfA_f2 | AAGCAGTGGCTTGCTAACCT |
| P8 | qPCR_atfA_r2 | GCATGTTGTACGGGTGAGAC |
| P9 | qPCR_terA_f3 | GGCCAGGTGAAAGAATTGAT |
| P10 | qPCR_terA_r3 | GACGATTGCTAGGAACCCAT |
| P11 | qPCR_terB_r2 | CCTGGATGGTAGAGAATGGG |
| P12 | qPCR_terB_r2 | CAGCCGACATCTGAATAACG |
| P13 | qPCR_terC_f | AATATCGAGTGGGCCGTATC |
| P14 | qPCR_terC_r | CCAGTAAATGGCTCTACGCA |
| P15 | qPCR_terR_f2 | GTCAATGCGCTCTACAGTCC |
| P16 | qPCR_terR_r2 | TGAAGATGGCCTCGTGATAC |
| P17 | qPCR_hapX_f1 | CCGCTCATCTTTGTCGAATA |
| P18 | qPCR_hapX_r1 | TAAAGGCATCCTCAATGCAC |
| P19 | qPCR_sreA_f2 | GCGTCGGTCCCTACTACTCT |
| P20 | qPCR_sreA_f2 | GAGGTTGAGCTCTGTGACTCC |
| P21 | qPCR_AnhapX_f1 | AGCCACACCACTCACCCTAT |
| P22 | qPCR_AnhapX_r1 | GCCCATTACACTAGCTGCCT |
| P23 | qPCR_fetC_f1 | GTCTGTGCTGCAACTGGAAC |
| P24 | qPCR_fetC_r1 | CATGTGATCGAGATCAACCC |
| P25 | qPCR_freB_f1 | GCCAGACCGGAAATTAGAGA |
| P26 | qPCR_freB_r1 | ATATCCCGAGTGAAGGGTTG |
| P27 | qPCR_ftrA_f1 | TATGGCTCGGTCATCTCGTA |
| P28 | qPCR_ftrA_r1 | TTCATCAGCACTCCAACTCC |
| P29 | qPCR_sidA_f1 | CACCAACTACAGCGTCGTG |
| P30 | qPCR_sidA_r1 | CTCGAGGACCTCCTTGGC |
| P31 | qPCR_sidC_f1 | CAGGTCAAGAACCTCAGCAA |
| P32 | qPCR_sidC_r1 | TCGATATCGGTGAATGAAGC |
| P33 | qPCR_sidD_f2 | GTCCTATGGCGTTGTCATTG |
| P34 | qPCR_sidD_r2 | TGCGAGATACGCAGGATTAG |
| P35 | qPCR_sidI_f1 | GCATTCCGGACCAGAAGTA |
| P36 | qPCR_sidI_r1 | CCTCCTGTCCAAACACAAAG |
| P37 | qPCR_mirA_f2 | TGGGTGTGTTGCTCTACCTC |
| P38 | qPCR_mirA_r2 | CCGATACTCATCGAGGCAA |
| P39 | qPCR_mirB_f1 | CTCCCTGCCTCTGAACTCTC |
| P40 | qPCR_mirB_r1 | CCAAATAAACGACAGAGCCA |
| P41 | qPCR_mirC_f1 | GTCTGCTGAATGTCCCTGTC |
| P42 | qPCR_mirC_r1 | CGGCAAATATTCCCGTAACT |
| P43 | qPCR_mirD_f2 | GACATCGGCTACATCGTCAT |
| P44 | qPCR_mirD_r2 | ATACTTCGCCAGCTTCTTCG |
| P45 | qPCR_sitA_f1 | ACTGGAATCTTCCTCGCATC |
| P46 | qPCR_sitA_r1 | CGCTGTAGCGTATGTGAAGG |
| P47 | NotI_P00145_f | GCGGCCGCAATATTTGTGTGTCGAGAACC |
| P48 | BglII_P00145_r | AGATCTCATGGTGCTGTGATGAGAAGTTTG |
| P49 | pCRIV_AnPgpdA_f | CACTAAAGGGACTAGTTCACCACAAAAGTCAGACGGCGTAACC |
| P50 | atfA_AnPgpdA_r | AACCGCCGCGGACATTGTGATGTCTGCTCAAGCGGGGTAGC |
| P51 | atfA_OE_f | ATGTCCGCGGCGGTTGCGTCG |
| P52 | pCRIV_atfAT_r | AACCTGCAGGACTAGTCCAGAACCAGCGATCAGGTCG |
| P53 | KpnI_cpcAup_f | GTGAATTCGAGCTCGGTACCCGCTTAAGAAGTGCCTG |
| P54 | NotI_cpcAup_r | CGTAATCAAGCGGCCGCGACGATGACGAGTAGGATCG |
| P55 | NotI_cpcAdn_f | GTATAATACGCGGCCGCCTTGACGAGTATTGTTATGACC |
| P56 | KpnI_cpcAdn_r | CTAGAGGATCCCCGGGTACCATCTGTTCTTTGCCCGCAG |
| P57 | KpnI_rhbAup_f | GTGAATTCGAGCTCGGTACCGACTGCTGCTGCGATAAC |
| P58 | NotI_rhbAup_r | CGTAATCAAGCGGCCGCTTCTCAAGGCAGCGTGTC |
| P59 | NotI_rhbAdn_f | GTATAATACGCGGCCGCTGAGAATGGAGGGACG |
| P60 | KpnI_rhbAdn_r | CTAGAGGATCCCCGGGTACCAAACAATTAGAGAACCAAGG |
| P61 | SmaI_areAup_for | CCCGGGACGTTGAATACCGGTTGG |
| P62 | NotI_areAup_rev | CGTGGAGCGGCCGCCGTAGACGAGGAGGGTG |
| P63 | NotI_areAdn_for | GTCTACGGCGGCCGCTCCACGGTGATGAGTATG |
| P64 | SmaI_areAdn_rev | CCCGGGAGAGGTGTGTTGTGAGG |
| P65 | pUC19_areAup_for | CGGCCAGTGAATTCGAGCTCGAGATGAGAAGGATCACTACTTC |
| P66 | pUC19_KpnAftAup_for | GTGAATTCGAGCTCGGTACCCAGAAAATTCCTGTCATCG |
| P67 | ptrA_NotIAftAup_rev | CGTAATCAAGCGGCCGCAGAAGTGGTGGTTGTGG |
| P68 | ptrA_NotIAftAdn_for | GTATAATACGCGGCCGCTGTGTTTTTATACTCTGATTGC |
| P69 | pUC19_KpnAftAdn_rev | CTAGAGGATCCCCGGGTACCCTGATATTCCAATCCACG |
| P70 | ble_NotITatfA_rev | TTGTGGTGAGCGGCCGCACCTTCCGGTGATTCAACC |
| P71 | ble_NotIatfAdn_f | CCCTCACTCGCGGCCGCTGTGTTTTTATACTCTGATTGCTTCC |
| P72 | pUCEcoIatfAup_F | GACGGCCAGTGAATTCCAGAAAATTCCTGTCATCGTCACCGTG |
| P73 | AnPgpdA_atfAup_R | TGACTTTTGTGGTGAAGAAGTGGTGGTTGTGGTCGACG |
| P74 | terRT_atfAdn_f | AACCTGACTACCATGGCGGCCGCTGTGTTTTTATACTCTGATTGC |
| P75 | pUCEcoIatfAdn_R | GGATCCCCGGAATTCCTGATATTCCAATCCACGGAGAC |
| P76 | AnPgpdA_for | TCACCACAAAAGTCAGACGGCGTAAC |
| P77 | terRT_rev | CATGGTAGTCAGGTTGTGC |
| P78 | AthapXup_r2 | CACGAGCTTCCTCAGGAAGC |
| P79 | degAthapX_C_r2 | RTCRAARTANCKNARNACNCCCAT |
| P80 | AreAZf663f | AACTGGGATCCAAGAACGGAGAGCAGAATG |
| P81 | AreAZf797r | ACGTAAGCTTTTAAGACGTAGTCGCCGCTGAGC |
| P82 | pUC_hapXup2_f | GTGAATTCGAGCTCGGTACCATCCACGTCCGAAGCC |
| P83 | ptrA_hapXup2_r | CGTAATCAAGCGGCCGCGATTCGTCCGAGC |
| P84 | ptrA_hapXdn2_f | GTATAATACGCGGCCGCCAGACCCAGAGGAGATG |
| P85 | pUC_hapXdn2_r | CTAGAGGATCCCCGGGTACCCGGCCCGTTGG |
| P86 | AnhapXup_hapXup_r | TGAAGGCGTCTCTCGGATTCGTCCGAGCTTTTATTCGAC |
| P87 | ble_hapXdn_f | CCCTCACTCGCGGCCGCCAGACCCAGAGGAGATGGAG |
| P88 | AnhapX_f | CGAGAGACGCCTTCATTGG |
| P89 | ble_AnhapX_r | TTGTGGTGAGCGGCCGCCCAGGTCCTATTCTAAAGACC |
| P90 | pUC19_sreAup2 | GTGAATTCGAGCTCGGTACCATCTTCTTCCTCTTCCCATC |
| P91 | ptrA_sreAup_r | CGTAATCAAGCGGCCGCCTGGGGGATGGATGC |
| P92 | ptrA_sreAdn_f | GTATAATACGCGGCCGCATTGACGATCGATTTTTCTACTCTAATC |
| P93 | pUC19_sreAdn2 | CTAGAGGATCCCCGGGTACCGGATCAAGGCTTCTCAAG |
| P94 | pUC_sreAup_f | GTGAATTCGAGCTCGGTACCGGGATACACTATTGACCAC |
| P95 | ble_NotITsreA_rev | TTGTGGTGAGCGGCCGCCAGCGCTGAAATAAACG |
| P96 | ble_NotIsreAdn_f | CCCTCACTCGCGGCCGCATTGACGATCGATTTTTCTACTCTAATC |
| P97 | pUC_sreAdn_r | CTAGAGGATCCCCGGGTACCAATCAAAGAAACCCTCATTCTAC |
| P98 | pUC_Kpn_sidAup_f | GTGAATTCGAGCTCGGTACCTAAGGTATCGACTC |
| P99 | ptrA_Not_sidAup_r | CGTAATCAAGCGGCCGCGATAGCTCTGGGAGC |
| P100 | ptrA_Not_sidAdn_f | GTATAATACGCGGCCGCTAAGGTAGGTACTGTAGG |
| P101 | pUC_Kpn_sidAdn_r | CTAGAGGATCCCCGGGTACCGCAGGTACAATACACTC |
| P102 | pUCHind_sidA_f | GCAGGCATGCAAGCTTTAAGGTATCGACTCTGGTAATC |
| P103 | ptrA_sidAT_rev | CGTAATCAAGCGGCCGCGGAGGTCCTACCTCAGCTATC |
| P104 | pUCHind_sidA_r | TGATTACGCCAAGCTTGCAGGTACAATACACTCCGTAG |
| P105 | ptrA_sidAdn_for | GTATAATACGCGGCCGCTAAGGTAGGTACTGTAGGTACAG |
| P106 | LacZ_up_down | GGCGTTACCCAACTTAATCGC |
| P107 | LacZ_mitte_up | CTCATCCATGACCTGACCATG |
| P108 | atfA_for1 | CCAACAAGAGAGCAAAGACG |
| P109 | atfA_rev1 | GTTGCATTCCCATATTGCCAGG |
| P110 | hapXup_for | CGTCTGAATCTGATATCTGTTCG |
| P111 | hapX_r | CTGGCTCTGTACGTTGTCC |
| P112 | hapXdn_rev | CCTACCGCGGCATCATCG |
| P113 | hapX_f1 | CCGCTCATCTTTGTCGAATA |
| P114 | pJET1.2_for | CGACTCACTATAGGGAGAGCGGC |
| P115 | pJET1.2_rev | AAGAACATCGATTTTCCATGGCAG |
| P116 | AthapXin_f2 | CATCCTTGACGCTCTCCTGC |
| P117 | AthapXin_f3 | GGTTGTTGCGGCGGTAAAGG |
