# Supplementary material for: Phytotoxin production in Aspergillus terreus is regulated by independent environmental signals
Source: eLife. 2015 Jul 14;4:e07861. doi: 10.7554/eLife.07861 (PMC4528345; doi:10.7554/eLife.07861)
Supplement: Figure 5—source data 1. — DOI: http://dx.doi.org/10.7554/eLife.07861.018 [file elife-07861-fig5-data1.doc]

**Figure 5 – Source Data 1. Analytical data of coprogen.**

^1^H NMR (500 MHz, DMSO-d_6_): δ = 9.84 (s, b, 3H, NO**H**), 8.27 (d, 1H, *J* = 7.3 Hz, AcN**H**), 8.12 (s, 2H, N**H**), 6.21 (s, 3H, H-4, H-16, H-26), 4.55 (s, b, 2H, OH), 4.15 (m, 3H, H-19, H‑21), 3.80 (m, 2H, H-9, H-11), 3.52 (t, 4H, *J* = 6.7 Hz, H-1, H-29), 3.49 (m, 6H, H-6, H-14, H-24), 2.38 (t, 2H, *J* = 6.6 Hz, H-18), 2.22 (t, 4H, *J* = 6.6 Hz, H-2, H-28), 2.01 (s, 9H, H‑3´. H-17´, H-27´), 1.83 (s, 3H, H-31) 1.73-1.47 (m, 12H, H-7, H-8, H-12, H-13, H-22, H‑23) ppm. ^13^C NMR (125 MHz, DMSO-d_6_): δ = 172.1 (C-20), 169.6 (C-30), 167.9 (C-10, C‑10´), 166.6 and 166.2 (C-5, C-15, C-25), 150.9 (C3, C-27), 148.6 (C-17), 117.2 (C-16), 116.3 (C-4, C-26), 62.3 (C-19), 59.2 (C-1, C-29), 53.8 and 53.7 (C-9, C-11), 52.0 (C-21), 46.8 and 46.4 (C-6, C-14, C-24), 43.8 (C-2, C-28), 39.0 (C-18), 30.3 (C-23), 28.0 (C-22), 23.1 (C‑7, C-13), 22.1 (C-31), 22.1 (C-8, C-12), 18.2 (C-3´, C-27´), 18.0 (C-17´) ppm. MS (ESI^+^): *m/z* 769 [M+H]^+^ (100%). HRMS calcd. for C_35_H_57_N_6_O_13_ [M+H]^+^: 769.3978, found 769.3975.
