# Supplementary material for: Phytotoxin production in Aspergillus terreus is regulated by independent environmental signals
Source: eLife. 2015 Jul 14;4:e07861. doi: 10.7554/eLife.07861 (PMC4528345; doi:10.7554/eLife.07861)
Supplement: Figure 5—source data 2. — DOI: http://dx.doi.org/10.7554/eLife.07861.019 [file elife-07861-fig5-data2.doc]

**Figure 5 – Source Data 2. Analytical data of ferrichrysin.**

MS (ESI^-^): *m/z* 746 [M-H]^-^ (100%). HRMS calcd. for C_29_H_48_N_9_O_14_ [M-H]^-^: 746.3342, found 746.3326.
